# Licochalcone E, a β-Amyloid Aggregation Inhibitor, Regulates Microglial M1/M2 Polarization via Inhibition of CTL1-Mediated Choline Uptake

**DOI:** 10.3390/biom13020191

**Published:** 2023-01-17

**Authors:** Eisuke Muto, Toshio Okada, Tsuyoshi Yamanaka, Hiroyuki Uchino, Masato Inazu

**Affiliations:** 1 Department of Anesthesiology, Tokyo Medical University, 6-7-1 Nishishinjuku, Shinjuku-ku, Tokyo 160-0023, Japan; 2 Department of Molecular Preventive Medicine, Tokyo Medical University, 6-1-1 Shinjuku, Shinjuku-ku, Tokyo 160-8402, Japan; 3 Institute of Medical Science, Tokyo Medical University, 6-1-1 Shinjuku, Shinjuku-ku, Tokyo 160-8402, Japan

**Keywords:** licochalcone, microglia, choline transporter, M1/M2 polarization

## Abstract

Alzheimer’s disease (AD) is thought to be a series of neuroinflammatory diseases caused by abnormal deposits of amyloid-β (Aβ) and tau protein in the brain as part of its etiology. We focused on Aβ aggregation and M1 and M2 microglial polarity in microglia to search for novel therapeutic agents. It has been reported that the inhibition of choline uptake via choline transporter-like protein 1 (CTL1) in microglia preferentially induces M2 microglial polarity. However, the role of the choline transport system on the regulation of microglial M1/M2 polarity in AD is not fully understood. Licochalcones (Licos) A–E, flavonoids extracted from licorice, have been reported to have immunological anti-inflammatory effects, and Lico A inhibits Aβ aggregation. In this study, we compared the efficacy of five Licos, from Lico A to E, at inhibiting Aβ1-42 aggregation. Among the five Licos, Lico E was selected to investigate the relationship between the inhibition of choline uptake and microglial M1/M2 polarization using the immortalized mouse microglial cell line SIM-A9. We newly found that Lico E inhibited choline uptake and Aβ1-42 aggregation in SIM-A9 cells in a concentration-dependent manner, suggesting that the inhibitory effect of Lico E on choline uptake is mediated by CTL1. The mRNA expression of tumor necrosis factor (TNF-α), a marker of M1 microglia, was increased by Aβ1-42, and its effect was inhibited by choline deprivation and Lico E in a concentration-dependent manner. In contrast, the mRNA expression of arginase-1 (Arg-1), a marker of M2 microglia, was increased by IL-4, and its effect was enhanced by choline deprivation and Lico E. We found that Lico E has an inhibitory effect on Aβ aggregation and promotes polarity from M1 to M2 microglia via inhibition of the CTL1 function in microglia. Thus, Lico E may become a leading compound for a novel treatment of AD.

## 1. Introduction

Alzheimer’s disease (AD) is a neurodegenerative disease characterized by the extracellular aggregation of amyloid-β (Aβ)-peptide and the consequent intracellular aggregation of tau protein. The causes for these events are unknown, but microglial inflammatory response would be playing a key role. The current focus is on understanding the pathogenesis of AD by targeting several mechanisms, including abnormal tau protein metabolism and Aβ deposition, and the development of treatments that can suppress or modify the progression of AD is underway. In recent years, an increasing number of studies have recognized that neuroinflammation, primarily caused by activated microglia, is involved in the pathogenesis of AD [1,2,3,4,5]. Activated microglia, such as macrophages in peripheral tissues, exist in two polar states: the M1 phenotype, which is largely associated with the release of inflammatory cytokines, and the anti-inflammatory M2 phenotype [6,7]. It has been suggested that during the early stages of AD, microglia with the M2 phenotype are activated and have a protective (anti-inflammatory) function that attempts to clear amyloid and release nerve growth factors [8]. The accumulation of Aβ and other toxins disrupts this process, leading to the activation of the proinflammatory M1 phenotype and the release of proinflammatory cytokines, causing self-proliferative neuronal damage. Some studies follow this bimodal change with PET studies [9]. Controlling the M1/M2 polarity of microglia may prevent the development of AD. Inhibiting the choline transport pathway has been reported to suppress the secretion of inflammatory cytokines by macrophages [10,11]. Thus, the choline transport system and cytokine release in microglia may be deeply involved.

Because choline is water-soluble, it must be taken up by the choline transporter in order to have various physiological effects in the cell. Choline is a biological factor deeply involved in cell growth and functions as a precursor of phospholipids such as phosphatidylcholine and sphingomyelin, which are major components of cell membranes [12]. Recent studies have shown that the choline transporter-like protein 1 (CTL1) is predominantly expressed in the mouse microglial immortalized cell line SIM-A9 and is strongly involved in choline transport [13]. Furthermore, the inhibition of CTL1-mediated choline uptake in SIM-A9 cells has been suggested to preferentially induce M2 microglial polarity [13]. CTL1 expressed in microglia is expected to be a therapeutic target molecule for inflammatory diseases such as AD with activation of the microglia.

**Figure 1:**
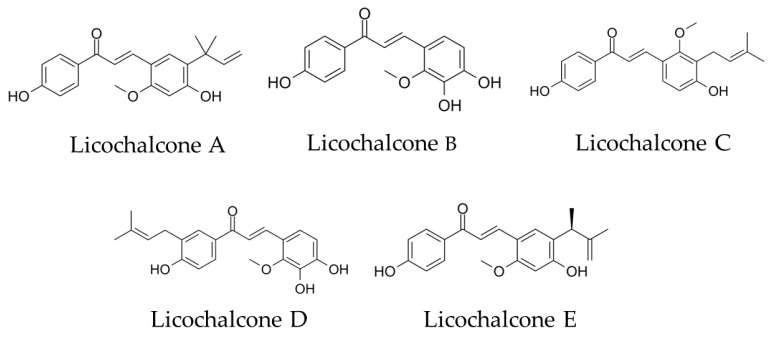
Chemical structural formula of licochalcones A, B, C, D and E.

We focused on the choline transporter and searched for candidates for AD treatment. The conditions for our search were (1) inhibition of Aβ aggregation and (2) inhibition of choline uptake in microglia. We hypothesized that the conversion to anti-inflammatory M2 polarity could be controlled by finding a new substance that inhibits the microglial choline transporter CTL1. Licochalcone A (Lico A), a flavonoid isolated from licorice, has been reported to inhibit Aβ aggregation [14]. Licos A–E (Figure 1) have been suggested to have immunological anti-inflammatory properties [15]. Therefore, we focused on the Lico family as candidates for CTL1 inhibitors.

In this study, we first compared the inhibitory effects of five types of Licos, A–E, on Aβ1-42 aggregation and choline uptake and selected Lico E. The aim of this study was to investigate how Lico E affects microglial polarity via the choline transporter function. Aβ1-42 promotes M1 polarity in microglia and secretes inflammatory cytokines, and interleukin-4 (IL-4) promotes M2 polarity in microglia and macrophages [16,17]. In this experiment, the mouse microglial cell line SIM-A9 was used to induce M1 polarity with Aβ1-42 and M2 polarity with IL-4, focusing on the effects of Lico E and choline uptake via CTL1 on each type of microglia.

## 2. Materials and Methods

### 2.1. Materials

Licos A, B, C and D were obtained from MedChem Express, Princeton, NJ, USA, and their purities were 99.58%, 99.41%, 99.55% and more than 99%. Lico E was obtained from Herbest (Baoji Herbest Bio-Tech Co., Ltd., Baoji, China), and its purity was over 99.17%. SensoLyte^®^ Thioflavin T Aβ1-42 Aggregation Kit and Aβ1-42 peptide were purchased from AnaSpec, Inc. (Fremont, CA, USA). The immortalized mouse microglia cell line SIM-A9 was obtained from Applied Biological Materials Inc. (Richmond, BC, Canada). RPMI 1640 medium and Penicillin-Streptomycin Solution were purchased from FUJI-FILM Wako Pure Chemical Corporation (Osaka, Japan). Choline-free RPMI 1640 medium was purchased from Cell Science & Technology Inst., Inc. (Sendai, Japan). CellTiter-Glo^®^ Luminescent Cell Viability Assay and Caspase-Glo^®^ 3/7 Assay were purchased from Promega Corporation (Madison, WI, USA). [^3^H]Choline and Hionic-Fluor were purchased from PerkinElmer Life Sciences, Inc. (Hopkinton, MA, USA). QIA shredder and RNeasy Mini Kit were purchased from Qiagen Inc. (Valencia, CA, USA). TaqMan^®^ Gene Expression Assays and TaqMan RNA-to-CT 1-Step Kit were purchased from Applied Biosystems (Foster City, CA, USA). Fetal bovine serum was purchased from Gibco (Grand Island, NY, USA).

### 2.2. Aβ1-42 Aggregation

The aggregation of Aβ1-42 was measured over time using the SensoLyte^®^ Thioflavin T Aβ1-42 Aggregation Kit. The SensoLyte^®^ ThT Aβ1-42 Aggregation Kit is a convenient and standard method for measuring Aβ42 aggregation using Thioflavin T (ThT) dye, and it was performed according to the manufacturer’s instructions. A 10 mM Lico (A–E) stock solution in 100% dimethyl sulfoxide (DMSO) was prepared and diluted to the desired concentration with 100% DMSO. The final DMSO concentration of Licos was set at 1%. To each well of a non-binding 96-well black microplate (Greiner Bio-One GmbH, Frickenhausen, Germany), 5 μL of 2 mM ThT, 0.5 μL of various concentrations of Licos and 44.5 μL of 250 μg/mL Aβ1-42 peptide were added. Assay buffer and 100% DMSO were used as vehicle controls. The plates were loaded into a FilterMax^TM^ F5 Multi-Mode Microplate Reader (Molecular Devices, LLC, Sunnyvale, CA, USA), and the fluorescence intensity was measured at 37 °C with Ex/Em = 440/484 nm and 15 s shaking between reads to facilitate aggregation. The fluorescence intensity was measured every 5 min for 60 min. The fluorescence reading from the blank control wells was used as the background fluorescence. Each value reported is the average of three readings for every sample. This background reading was subtracted from the readings of the other wells. All fluorescence readings are expressed in relative fluorescence units (RFU).

### 2.3. Cell Culture

SIM-A9 cells retain typical microglial characteristics and have been reported to respond to inflammatory stimuli similarly to primary microglia, particularly with respect to phagocytic activity and inflammatory signals in response to LPS and Aβ stimulation. Furthermore, LPS increased the levels of inducible nitric oxide synthase and cyclooxygenase-2, whereas IL-4 stimulation increased arg-1 levels, demonstrating that SIM-A9 cells can switch their profiles to pro- or anti-inflammatory phenotypes, respectively [18]. SIM-A9 cells exhibit key characteristics of cultured primary microglia and serve as a valuable model system for the investigation of microglial behavior in vitro.

Cells were cultured in RPMI 1640 medium supplemented with 10% fetal bovine serum (Gibco, Grand Island, NY, USA) and Penicillin-Streptomycin Solution (FUJI-FILM-Wako) using non-coated flasks and 24-well plates. The medium was replaced every 3–4 days and incubated at 37 °C in a humidified atmosphere of 5% CO_2_ and 95% air.

### 2.4. Measurement of Cell Number

Cell counts were measured as previously described [19]. SIM-A9 cells were seeded in 24-well plates at 5000 cells/well, cultured for 24 h and then treated with Licos D and E for 24 h. The cell number was determined using the CellTiter-Glo^®^ Luminescent Cell Viability Assay. Chemiluminescence measurements were performed using a Filter-Max^TM^ F5 Multi-Mode Microplate Reader (Molecular Devices, LLC, Sunnyvale, CA, USA).

### 2.5. Measurement of Caspase-3/7 Activity

Caspase-3/7 activity assays were performed as previously described [19,20]. Cells were cultured in 48-well plates for 48 h and then treated with Lico D and E for 24 h. Caspase-3/7 activity and cell number were simultaneously measured using the Caspase-Glo^®^ 3/7 Assay and the CellTiter-Glo^®^ Luminescent Cell Viability Assay. The Caspase-Glo^®^ 3/7 Assay is a homogeneous, luminescent assay that measures both caspase-3 and -7 activities. The assay provides a luminogenic caspase-3/7 substrate, which contains the tetrapeptide sequence DEVD, in a reagent optimized for caspase activity and luciferase activity. Luminescence was measured with a FilterMax F5 Multi-Mode Microplate Reader (Molecular Devices, LLC, Sunnyvale, CA, USA). Caspase-3/7 activity was calculated as the activity per number of cells.

### 2.6. [^3^H]Choline Uptake into SIM-A9 Cells

With reference to earlier studies [19,21], [^3^H]choline uptake analysis was carried out using [^3^H]choline (specific activity: 2800 GBq/mmol). SIM-A9 cells were cultured for 48 h using a non-coated 24-well culture plate. The cells were washed twice with 1 mL of uptake buffer and preincubated with each concentration of Licos D and E for 20 min. Uptake buffer was used to wash the cells twice. Next, [^3^H]choline (final conc. = 10 µM) was added and taken up for 20 min at 37 °C. The uptake buffer was then discarded and quickly washed three times with an ice-cold uptake buffer to terminate uptake. Aliquots of cells dissolved in 0.1% Triton X-100 were mixed with the liquid scintillation cocktail Hionic-Fluor, and radioactivity was measured with a liquid scintillation counter (Tri-Carb^®^ 2100 TR, Packard, Meriden, CT, USA). Specific uptake of [^3^H]choline was defined as the difference in total [^3^H]choline uptake in the presence and absence of 30 mM unlabeled choline chloride.

### 2.7. RNA Extraction and Real-Time PCR Assay

Aβ1-42 peptide was purchased from AnaSpec, Inc. (Fremont, CA, USA). An amount of 1 mM Aβ1-42 was dissolved in assay buffer (AnaSpec, Inc., Fremont, CA, USA), and the fibril formation of Aβ1-42 was prepared by shaking vigorously for 15 s at 5 min intervals for 8 h at 37 °C.

The mRNA expression levels of the target genes were quantified according to previously established methods [20,22,23]. Total RNA was isolated from SIM-A9 cells after various stimuli using a QIA shredder and RNeasy Mini Kit according to the manufacturer’s instructions. TaqMan^®^ Gene Expression Assays (Applied Biosystems, Foster City, CA, USA; Thermo Fisher Scientific, Inc. Waltham, MA, USA) were used to select TaqMan probes corresponding to target mouse mRNAs (TNF-α, ARG1 and housekeeping gene β-actin). The accession numbers of the target genes and the assay IDs of the Taq-Man probes are TNF-α: NM_001278601, Mm00443258_m1; Arg-1: NM_001199186.1, Mm00475988_m1; and β-actin: AK078935.1, Mm00607939_s1. Data from one-step real-time PCRs conducted using the TaqMan RNA-to-CT 1-Step Kit (Applied Biosystems) were analyzed using the Light Cycler 96 System (Roche Diagnostics, Mannheim, Germany). Relative mRNA expression levels of target genes were computed using the comparative cycle time method, and the expression levels of target genes were computed relative to β-actin.

### 2.8. Data Analysis

All the data are presented as the mean ± standard deviation (SD). Statistical analyses were performed with Dunnett’s multiple comparisons test and Šídák’s multiple comparisons test using the statistical analysis software GraphPad Prism 9 (GraphPad, San Diego, CA, USA). Statistics with *p*-values under 0.05 were considered significant. IC_50_ values were calculated by the non-linear regression of the data using the four-parameter logistic equation.

## 3. Results

### 3.1. Licochalcones A, B, C, D and E Inhibit Aβ1-42 Aggregation In Vitro

**Figure 2:**
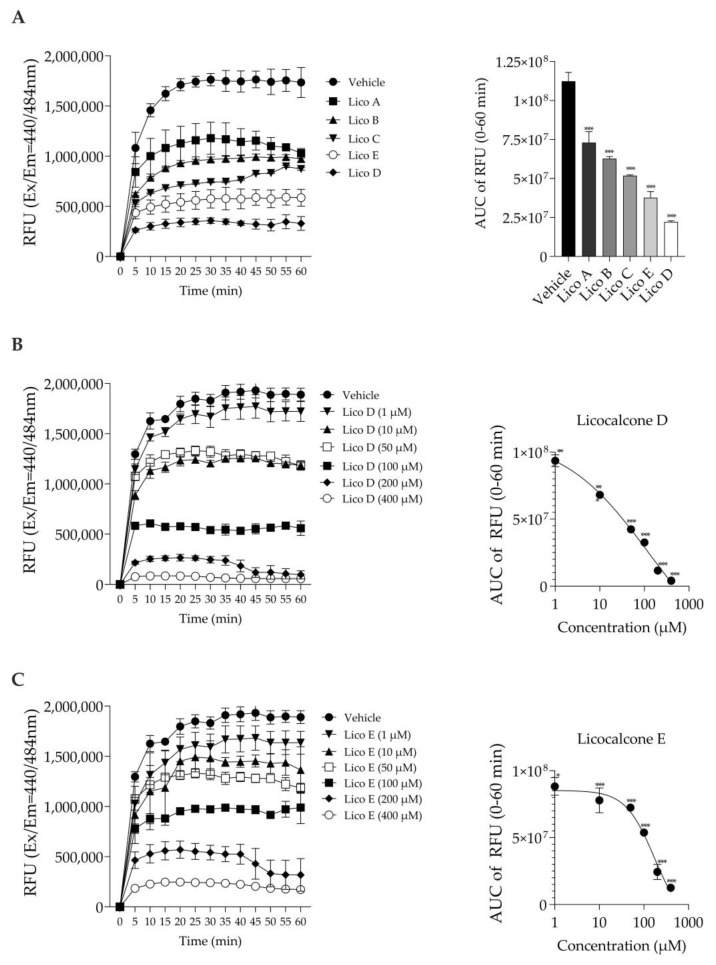
Effect of Licos A, B, C, D and E on Aβ1-42 aggregation. The right-side figure is the AUC of the left-side figure. (**A**) The aggregation of Aβ1-42 (213 µg/mL) in the absence or presence of Licos. The concentration of substances was 100 μM. (**B**) The aggregation of Aβ1-42 in the absence or presence of Lico D. (**C**) The aggregation of Aβ1-42 in the absence or presence of Lico E. The AUC data were fitted to non-linear regression analysis. Values are the mean ± SD (*n* = 3 samples/group). * *p* < 0.05, ** *p* < 0.005 and *** *p* < 0.0005 vs. vehicle control. Statistical analysis of the data was performed using Dunnett’s multiple comparisons test.

The inhibitory effect on Aβ1-42 aggregation was measured based on the fluorescence generated by thioflavin T binding [24]. First, we compared the Aβ1-42 aggregation inhibitory effects of each of the five Licos A–E substances at a concentration of 100 µM. As shown in Figure 2A, all licochalcones (Licos A–E) significantly inhibited the aggregation of Aβ1-42 compared to the vehicle control in the order of D > E > C > B > A. In particular, we found that Licos D and E at various concentrations significantly inhibited the aggregation of Aβ1-42 in a concentration-dependent manner (Figure 2B,C). The IC_50_ values of Licos D and E calculated from the AUC data were 30.4 and 103.7 µM, respectively. Licos A–E at 100 µM showed no effect on the relative fluorescence intensity at Ex440 nm/Em484 nm (Figure S1). Furthermore, we acquired UV-vis spectra of the 100 µM Lico A-E. Licos A-E had no absorption at 440 and 484 nm (Figure S2). It was concluded that Licos A-E did not affect the measurement system of Aβ1-42 aggregation.

### 3.2. Lico E Exhibits Lower Cytotoxicity than Lico D in SIM-A9 Cells

**Figure 3:**
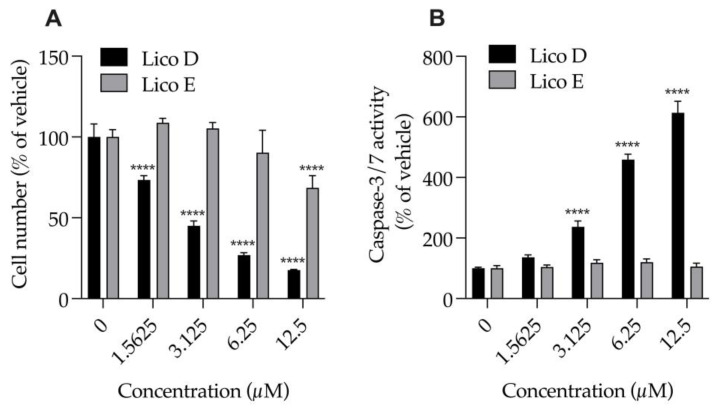
Effect of Licos D and E on the number of cells and caspase-3/7 activity of SIM-A9 cells. (**A**) The number of viable cells was determined after each separate treatment with Licos D and E (1.5625, 3.125, 6.25 and 12.5 μM) for 24 h. Cell number was calculated using 100% viable cells in the vehicle control group. (**B**) Caspase-3/7 activity was presented as a percentage of vehicle control. Values are the mean ± SD (*n* = 4). **** *p* < 0.0001 compared with vehicle control. Statistical analysis of the data was performed using Tukey’s multiple comparisons test.

We examined the effects of Licos D and E (1.6, 3.25, 6.5 and 12.5 μM), which had strong inhibitory effects on Aβ1-42 aggregation, on the numbers of cells and caspase-3/7 activity of SIM-A9 cells (Figure 3). Treatment with Lico D for 24 h significantly and strongly reduced the number of cells (EC_50_ = 2.9 µM) and significantly increased caspase-3/7 activity in a concentration-dependent manner, and both effects were inversely correlated. At 2.9 µM, the 50% effective concentration of Lico D that causes cell death, caspase-3/7 activity increases to 221.1%. On the other hand, when treated with Lico E for 24 h, a decrease in cell number of about 30% was observed only at 12.5 µM. Treatment of Lico E up to 12.5 µM for 24 h had no effect on caspase-3/7 activity. Lico E was found to be less cytotoxic to SIM-A9 cells than Lico D.

### 3.3. Licos D and E Inhibit Choline Uptake at Concentrations That Do Not Increase Membrane Permeability

**Figure 4:**
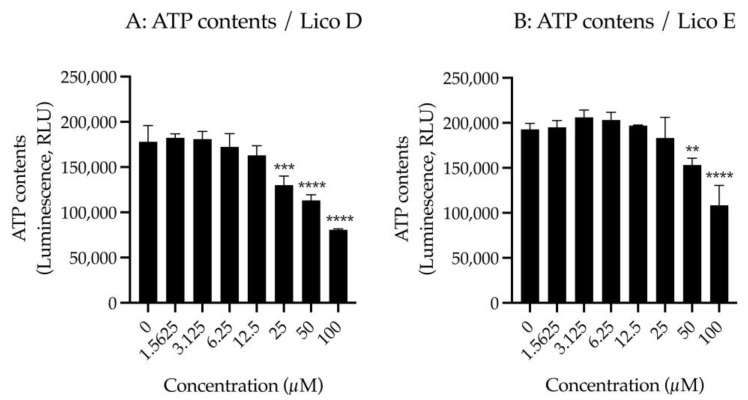

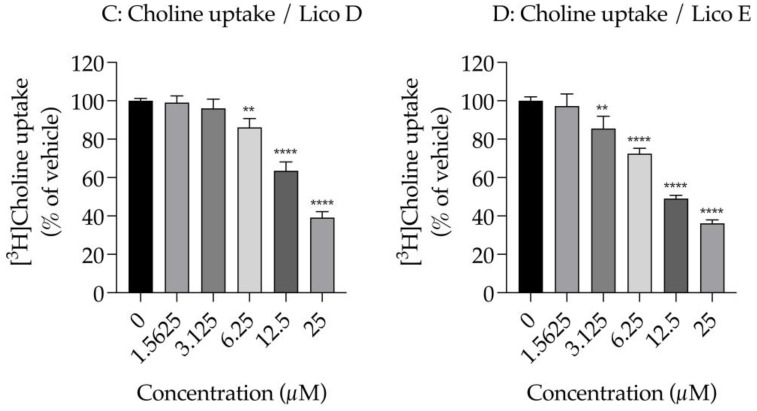
Effects of Licos D and E on membrane permeability and [^3^H]choline uptake in SIM-A9 cells. ATP contents were measured after treatment with 1.5625–100 µM of Licos D (**A**) and E (**B**) for 40 min. ATP contents were determined using the CellTiter-Glo^®^ Luminescent Cell Viability Assay. After preincubation of Licos D (**C**) and E (**D**) for 20 min, 10 µM [^3^H]choline was added and uptake was measured for 20 min. IC_50_ values of Licos D and E for inhibition of [^3^H]choline uptake were 17.1 and 12.4 μM, respectively. The results are presented as a percentage of uptake measured with the vehicle control. Values are the mean ± SD (*n* = 4). ** *p* < 0.005, *** *p* < 0.0005 and **** *p* < 0.0001 compared with vehicle control. Statistical analysis of the data was performed using Dunnett’s multiple comparisons test.

First, we examined the effect of Licos D and E on membrane permeability in SIM-A9 cells. Impaired membrane permeability induces cell death and, consequently, decreased ATP contents are observed. We, therefore, measured ATP contents after treatment with up to 100 µM of Licos D and E for 40 min in SIM-A9 cells. Licos D and E were observed to impair membrane permeability and decrease ATP contents at high concentrations above 25 µM (Figure 4A,B). Based on these results, we investigated the effect of Licos D and E on [^3^H]choline uptake at concentrations below 25 µM.

We examined the effects of Licos D and E on [^3^H]choline uptake in SIM-A9 cells since the inhibition of CTL1-mediated choline uptake promotes polarity from M1 to M2 microglia [13]. Both Licos D and E inhibited [^3^H]choline uptake in a concentration-dependent manner, with IC_50_ values of 17.1 and 12.4 μM, respectively (Figure 4B,C).

### 3.4. Choline Deficiency Suppresses Aβ1-42-Stimulated Increase in TNF-a mRNA Expression and Enhances IL-4-Stimulated Increase in Arg-1 mRNA Expression

First, we examined the mRNA expression of M1 and M2 markers by 4 h stimulation of Aβ1-42 and IL-4 and found that the mRNA expression of the M1 microglial marker TNF-α and the M2 microglial marker Arginase-1 (Arg-1) was significantly increased (Figure S3). Therefore, changes in the polarity of M1 and M2 microglia were assessed by changes in TNF-α and Arg-1 mRNA expression, respectively.

**Figure 5:**
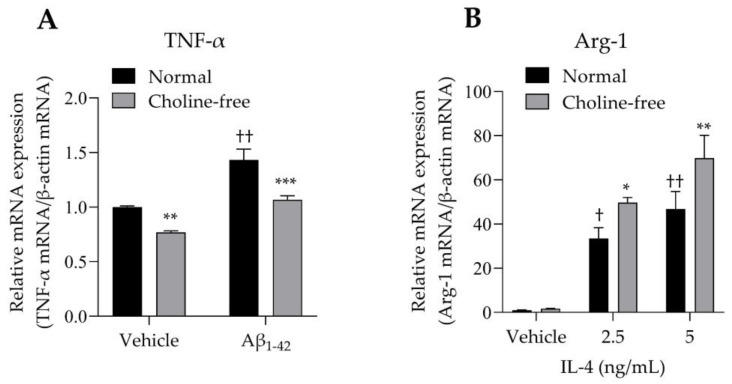
Effect of choline deficiency on TNF-α and Arg-1 mRNA expression in SIM-A9 cells stimulated by Aβ1-42 and IL-4. (**A**) Expression of TNF-α mRNA in SIM-A9 cells stimulated with 10 μM Aβ1-42 in normal or choline-free medium for 4 h. Values are the mean ± SD (*n* = 3). ** *p* < 0.005 and *** *p* < 0.0005 compared with normal culture medium under the same condition. †† *p* < 0.0001 compared with the vehicle of normal culture medium. Statistical analysis of the data was performed using Šídák’s multiple comparisons test. (**B**) Expression of Arg-1 mRNA in SIM-A9 cells stimulated with IL-4 (2.5 and 5 ng/mL) in normal or choline-free medium for 4 h. Values are the mean ± SD (*n* = 3). * *p* < 0.05 and ** *p* < 0.005 compared with normal culture medium under the same condition. Statistical analysis of the data was performed using Šídák’s multiple comparisons test. † *p* < 0.01 and †† *p* < 0.0001 compared with the vehicle of normal culture medium. Statistical analysis of the data was performed using Dunnett’s multiple comparisons test. Relative expression is expressed as a ratio of the target mRNA to β-actin mRNA.

The mRNA expression levels of TNF-α and Arg-1 in SIM-A9 cells stimulated with Aβ1-42 or IL-4 were analyzed. SIM-A9 cells stimulated with 10 μM Aβ1-42 for 4 h showed a significant increase in TNF-α mRNA levels under normal conditions (Figure 5A). These enhancing effects were markedly suppressed by choline deficiency (Figure 5A). Choline deficiency also significantly suppressed basal TNF-α mRNA expression. Stimulation of SIM-A9 cells with IL-4 significantly increased Arg-1 mRNA in a concentration-dependent manner under normal conditions (Figure 5B). These potentiating effects were markedly enhanced by choline deficiency (Figure 5B).

### 3.5. Lico E Suppresses TNF-α mRNA Expression and Enhances Arg-1 mRNA Expression in SIM-A9 Cells Stimulated with Aβ1-42 and IL-4

**Figure 6:**
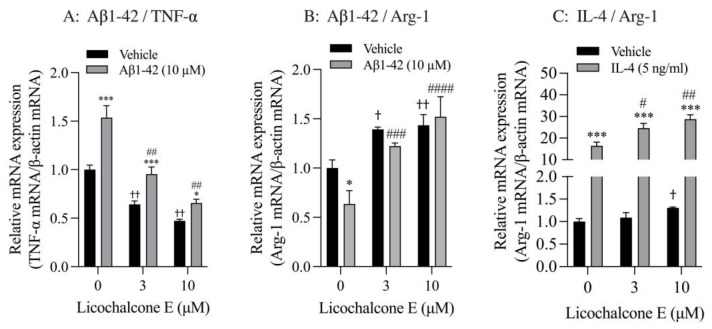
Effect of Lico E on TNF-α and Arg-1 mRNA expression in SIM-A9 cells stimulated with Aβ1-42 and IL-4. (**A**) Effect of Lico E on TNF mRNA expression in SIM-A9 cells stimulated with Aβ1-42. (**B**) Effect of Lico E on Arg-1 mRNA expression in SIM-A9 cells stimulated with Aβ1-42. Cells were treated with various concentrations (0, 3 and 10 μM) of Lico E for 30 min, followed by stimulation with 10 μM Aβ1-42 for 4 h. Values are the mean ± SD (*n* = 3). * *p* < 0.05 and *** *p* < 0.0005 compared with vehicle control. Statistical analysis of the data was performed using Šídák’s multiple comparisons test. † *p* < 0.05 and †† *p* < 0.0001 compared with vehicle control (0 µM of Lico E). Statistical analysis of the data was performed using Dunnett’s multiple comparisons test. # *p* < 0.005 and ## *p* < 0.0005, ### *p* = 0.0005 and #### *p* < 0.0001 compared with vehicle control (0 μM of Lico E). Statistical analysis of the data was performed using Dunnett’s multiple comparison test. (**C**) Effect of Lico E on Arg-1 mRNA expression in SIM-A9 cells stimulated with IL-4. Cells were treated with various concentrations (0, 3 and 10 μM) of Lico E for 30 min, followed by stimulation with 5 ng/mL IL-4 for 4 h. Values are the mean ± SD (*n* = 3). *** *p* < 0.0005 compared with vehicle control. Statistical analysis of the data was performed using Šídák’s multiple comparisons test. † *p* < 0.01 compared with vehicle control (0 µM of Lico E). Statistical analysis of the data was performed using Dunnett’s multiple comparisons test. # *p* < 0.005 and ## *p* < 0.0005 compared with vehicle control (0 μM of Lico E). Statistical analysis of the data was performed using Dunnett’s multiple comparisons test.

Finally, we analyzed the effect of Lico E on TNF-α and Arg-1 mRNA expression in SIM-A9 cells stimulated with Aβ1-42 and IL-4. SIM-A9 cells stimulated with 10 μM Aβ1-42 had significantly increased TNF-α mRNA levels compared with vehicle controls. These increased effects were significantly inhibited by Lico E treatment in a concentration-dependent manner (Figure 6A). Furthermore, Lico E significantly inhibited the expression of basal TNF-α mRNA in a concentration-dependent manner (Figure 6A). Stimulation with 10 μM Aβ1-42 significantly reduced Arg-1 mRNA levels compared with vehicle controls. Lico E increased Arg-1 mRNA expression, which was reduced by Aβ1-42 stimulation (Figure 6B). Lico E significantly increased basal Arg-1 mRNA expression in a concentration-dependent manner (Figure 6B).

SIM-A9 cells stimulated with 5 ng/mL IL-4 had significantly increased Arg-1 mRNA levels compared with vehicle controls (Figure 6C). These increased effects were significantly enhanced by Lico E treatment in a concentration-dependent manner (Figure 6C). In addition, 10 μM of Lico E significantly increased the expression of basal Arg-1 mRNA.

## 4. Discussion

AD is characterized by the abnormal aggregation of Aβ, forming extracellular fibrous deposits called amyloid plaques. The inhibition of Aβ aggregation is, therefore, expected to be a way to stop or slow the progression of AD [25]. In a recent report, ^11^C-(R)-PK11195-PET, which can detect microglial activation in patients with AD and mild cognitive impairment (MCI), revealed two peaks of microglia activity in disease progression [9]. The first peak, which corresponds to the stage of normal cognitive function before MCI, shows the properties of M2 microglia and is thought to suppress Aβ aggregation and its clearance. The second peak corresponds to a stage of cognitive decline, showing the properties of M1 microglia, which release inflammatory cytokines and induce neuropathy, resulting in cognitive dysfunction. It has been suggested that the inhibition or elimination of Aβ aggregation may indirectly maintain neuronal function. Microglia can exert neuroprotective effects by degrading Aβ plaques in response to the accumulation of Aβ [16]. Therefore, it is important to suppress the function of neuropathic M1 microglia and activate neuroprotective M2 microglia as a therapeutic strategy for AD.

Lico A, a flavonoid isolated from licorice, has been reported to inhibit Aβ1-42 aggregation [14]. The search for new derivatives of Lico A that prevent Aβ aggregation and oxidation and promote neuroprotection has been investigated [14]. Therefore, we focused on the licochalcone family (Licos A–E) and examined its effects on the inhibition of Aβ1-42 aggregation and changes in M1 and M2 microglia polarity. Among them, Licos D and E were found to have strong inhibitory effects on Aβ1-42 aggregation. When the effects of both compounds on the cell viability of SIM-A9 cells were examined, Lico D showed a stronger decrease in cell number compared to Lico E. A characteristic difference between the two compounds was that Lico D increased caspase-3/7 activity and induced cell death by apoptosis, whereas up to 12.5 µM Lico E had no effect. In terms of cytotoxicity, Lico E appears to be superior to Lico D as a lead compound. In the future, the cytotoxicity of Lico E needs to be investigated not only on microglia but also on neuronal cells, and the search for more safe derivatives than Lico E is needed.

In cancer cells, the CTL1-mediated choline uptake inhibitors Amb4269675, Amb4269951 and Amb544925 have been reported to inhibit cell proliferation at low concentrations and induce cell death at high concentrations [19,20,21]. Inhibiting CTL1-mediated uptake of choline is thought to decrease intracellular choline and decrease the synthesis of phosphatidylcholine, a component of the plasma membrane, resulting in the inhibition of cell proliferation. Furthermore, strong inhibition of CTL1-mediated choline uptake causes cell death by the induction of apoptosis via the ceramide/survivin pathway [19,20,21]. In the current study, although Licos D and E had similar inhibitory effects on choline uptake, the strong inhibition of cell survival by Lico D may be due to a mechanism other than inhibition of choline uptake. Lico D has been reported to have various biological activities, such as antioxidant, anti-inflammatory and anticancer properties [15]. Lico D inhibits the phosphorylation of NF-κB p65 in the LPS signaling pathway [26]. Lico D also inhibits JAK2, EGFR and Met (c-Met) activities and induces ROS-dependent apoptosis [27]. These biological activities of Lico D may have suppressed the cell viability of SIM-A9 cells. Lico E has also been found to possess a variety of pharmacological profiles, including anticancer, antiparasitic, antibacterial, anti-inflammatory and antidiabetic effects. In particular, cell viability suppression and apoptosis induction were observed at high concentrations of 50 μM [28,29]. The IC_50_ value for inhibition of [^3^H]choline uptake by Lico E was 12.4 μM, which is close to the concentration of 12.5 µM that reduces the number of cells (about 30% inhibition), suggesting that the inhibition of choline uptake by Lico E caused inhibition of cell proliferation. Lico E does not show any effect on caspase-3/7 activity up to 12.5 µM, suggesting that it does not induce cell death by apoptosis.

Accumulating evidence suggests that microglia are major immune cells in the central nervous system and act as key players in the development of neurodegenerative diseases such as AD. It is now well known that microglia are functionally plastic and have a dual phenotype, the inflammatory M1 phenotype and the anti-inflammatory M2 phenotype. Therefore, suppressing the M1 phenotype and stimulating the M2 phenotype may be a potential therapeutic approach for neuroinflammation-related diseases such as AD. Recent studies have suggested that the inhibition of CTL1-mediated choline uptake in SIM-A9 cells suppresses the M1 phenotype and activates the M2 phenotype [13], and CTL1 expressed in microglia has attracted attention as a therapeutic target molecule for inflammatory diseases associated with microglial activation such as AD.

Aβ activates the M1 phenotype in microglia in vitro [16,30,31], and the microglial activity marker CD68 and the M1 phenotype markers TNF-α and interleukin-1β have been shown to be upregulated in Aβ-stimulated primary microglia [16,32]. TNF-α has been shown to play a pivotal role in the early inflammatory process of AD in both animal models of AD and in longitudinal human studies. In AD, TNF-α is chronically released from activated microglia, neurons and astrocytes, and its release is stimulated by increased levels of extracellular Aβ [33,34,35,36]. TNF-α stimulates γ-secretase activity, increases Aβ peptide synthesis and may further increase TNF-α release [37]. Animal studies have highlighted that blockade of the TNF-α pathway is associated with decreased histopathological markers, such as the formation of Aβ plaques and a decreased number of microglial cells in the AD brain [38]. In humans, studies have detected elevated levels of TNF-α in both MCI and AD dementia [38,39]. Against this background, we analyzed the effects of Lico E and CTL1 functions on the increased expression of TNF-α by Aβ stimulation, one of the M1 phenotypes of microglia. In this study, stimulation of SIM-A9 cells with Aβ1-42 significantly increased TNF-α mRNA expression. This increase in TNF-α mRNA expression was suppressed by choline deficiency and Lico E treatment. These findings suggest that Lico E inhibited CTL1-mediated choline uptake and consequently suppressed TNF-α mRNA expression. CTL1 inhibitors such as Lico E suppress the M1 phenotype of microglia and can be proposed as therapeutic target molecules for inflammatory brain diseases such as AD. Although these mechanisms are unclear, TNF-α is released from microglia in the form of encapsulated extracellular vesicles (EVs), small particles formed by a bilayer of phospholipids [40], suggesting that the inhibition of choline uptake in microglia may influence EV formation. In the future, it is necessary to examine whether EV formation is affected by CTL1-mediated inhibition of choline uptake. Very interestingly, we found that Aβ1-42 stimulation of SIM-A9 cells decreased the expression of the M2 marker Arg-1 mRNA, and Lico E suppressed the decrease in Arg-1 mRNA expression by Aβ1-42 stimulation. Furthermore, Lico E increased Arg-1 mRNA expression in the basal state and conversely suppressed TNF-α mRNA expression. These results suggest that Aβ1-42 activates the M1 phenotype and suppresses the M2 phenotype in microglia, and Lico E is expected to provide neuroprotection by reversing these changes.

IL-4 is well known for inducing anti-inflammatory M2 microglia/macrophages. In the periphery, IL-4 is produced from activated Th2 cells, is important for humoral immunity and antigen presentation and induces the class change of B cells to antibody-producing cells [41]. In the central nervous system, IL-4/IL-13 was found to shift the polarity of Aβ-induced microglia to the M2 phenotype. Induction of M2 microglia with IL-4 and IL-13 may promote Aβ degradation. In fact, previous studies showed that M2 microglia reduced the deposition of various types of Aβ (Aβ1-38, Aβ1-40 and Aβ1-42) both in vitro and in vivo [42,43]. Similar to the study by Okada et al. [13], we observed upregulation of Arg-1 mRNA expression by IL-4 stimulation in SIM-A9 cells. Interestingly, the increase in IL-4-stimulated Arg-1 mRNA expression was enhanced by choline deficiency and treatment with Lico E, which has an inhibitory effect on CTL1-mediated choline uptake. Inhibition of CTL1 function may stimulate the M2 phenotype of microglia and promote Aβ degradation.

This study is consistent with a previous study by Okada et al., which showed that choline deprivation or CTL1 inhibition suppressed mRNA expression of the proinflammatory cytokines IL-1β and IL-6 in LPS-stimulated M1 microglia and enhanced the mRNA expression of Arg-1 in IL-4-stimulated M2 microglia [13]. We propose that CTL1-mediated inhibition of choline uptake is a novel mechanism to promote the microglial phenotype from M1 to M2 and represents a new therapeutic strategy for AD treatment. In addition, Lico E appears to be a lead compound that enhances neuroprotection by promoting polarization from M1 to M2 microglia, thereby contributing to the delay or suppression of AD development.

This study provides novel findings indicating that Lico E promotes the polarity of microglia activated by Aβ1-42 from M1 to M2 via inhibition of the CTL1 function, but the use of only SIM-A9 cells is a major limitation. In the future, experiments using primary cultured microglia or human microglia and in vivo experiments in AD model animals will be necessary. Furthermore, not only the changes in TNF-α and Arg-1 mRNA, but also their protein levels need to be verified, as well as a comprehensive analysis of other M1/M2 markers.

In conclusion, we found that Lico E has an inhibitory effect on Aβ1-42 aggregation and promotes the polarity from M1 to M2 microglia via inhibition of CTL1 function in microglia. Thus, Lico E may become a lead compound for a novel treatment of AD.

## Data Availability

The materials described in the manuscript, including all relevant raw data, will be freely available to any scientist wishing to use them for non-commercial purposes upon request via e-mail to the corresponding author (M.I.).

## Supplementary Materials

The following supporting information can be downloaded at: https://www.mdpi.com/article/10.3390/biom13020191/s1, Figure S1: Effect of 100 µM Licos A–E on relative fluorescence intensity at Ex440 nm/Em484 nm. Figure S2: UV-vis spectra of 100 μM Licos A–E. Figure S3: Expression of mRNAs for M1 and M2 markers after stimulation with Aβ1-42 and IL-4 for 4 h.
