# Supplementary material for: Licochalcone E, a β-Amyloid Aggregation Inhibitor, Regulates Microglial M1/M2 Polarization via Inhibition of CTL1-Mediated Choline Uptake
Source: Biomolecules. 2023 Jan 17;13(2):191. doi: 10.3390/biom13020191 (PMC9953043; doi:10.3390/biom13020191)
Supplement: Supplementary file 1 [file biomolecules-13-00191-s001.zip › biomolecules-2059366-supplementary.pdf]

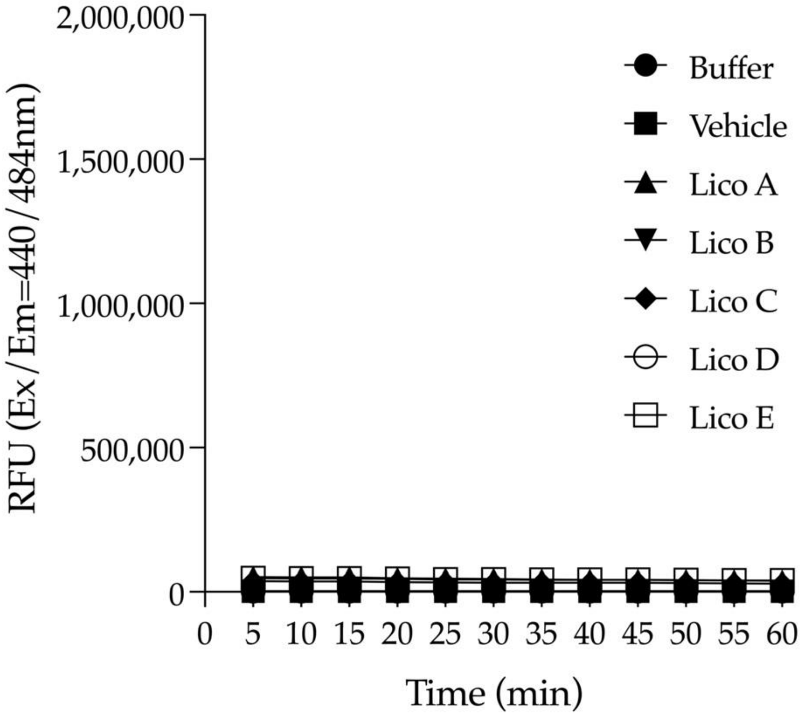

Figure S1. Effect of 100 μM Licos A-E on relative fluorescence intensity at Ex440nm/Em484nm.

Lico A (100 µM)

Lico B (100 µM)

Lico C (100 µM)

Lico D (100 µM)

Lico E (100 µM)

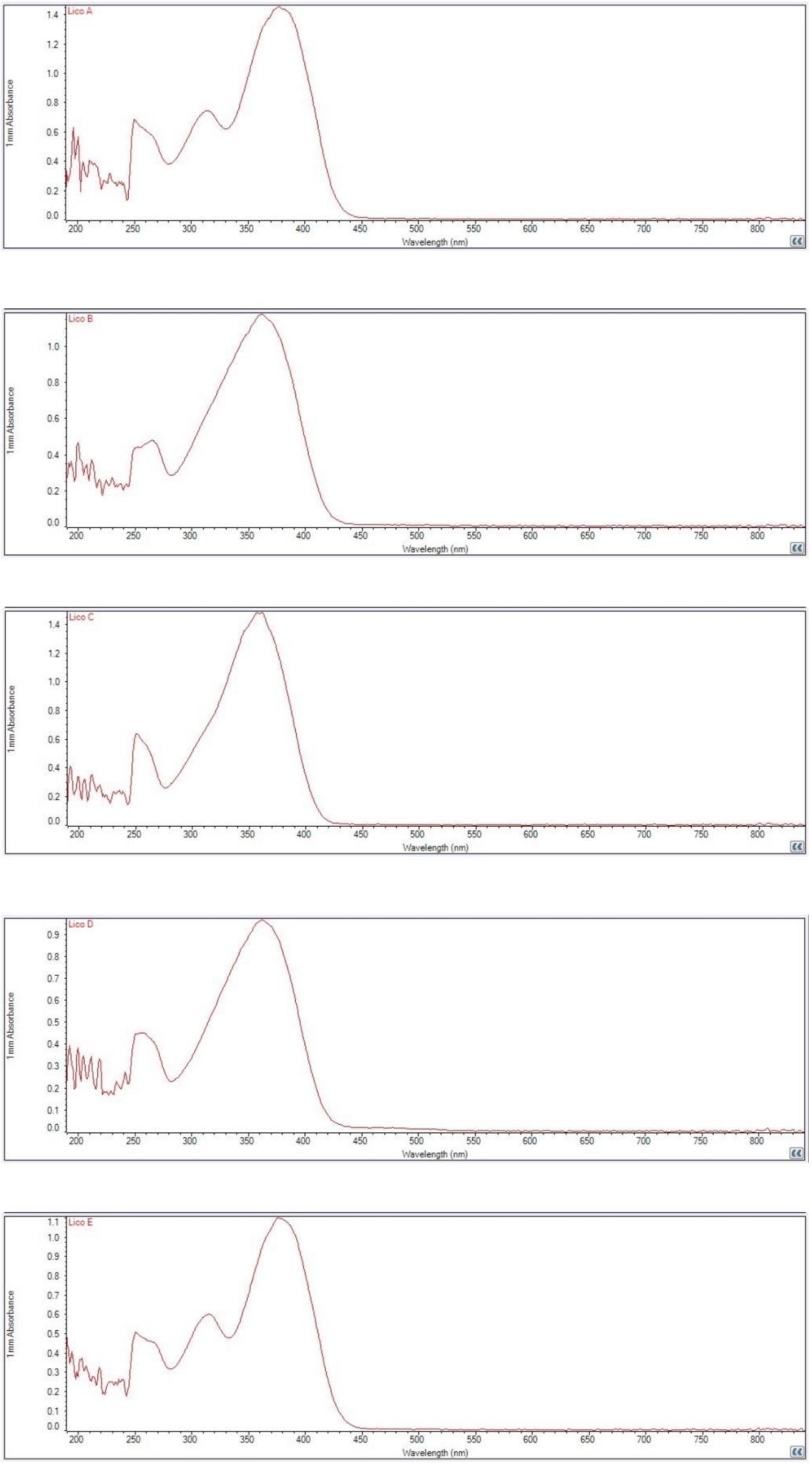

Figure S2. UV-vis spectra of 100 µM Licos A-E.

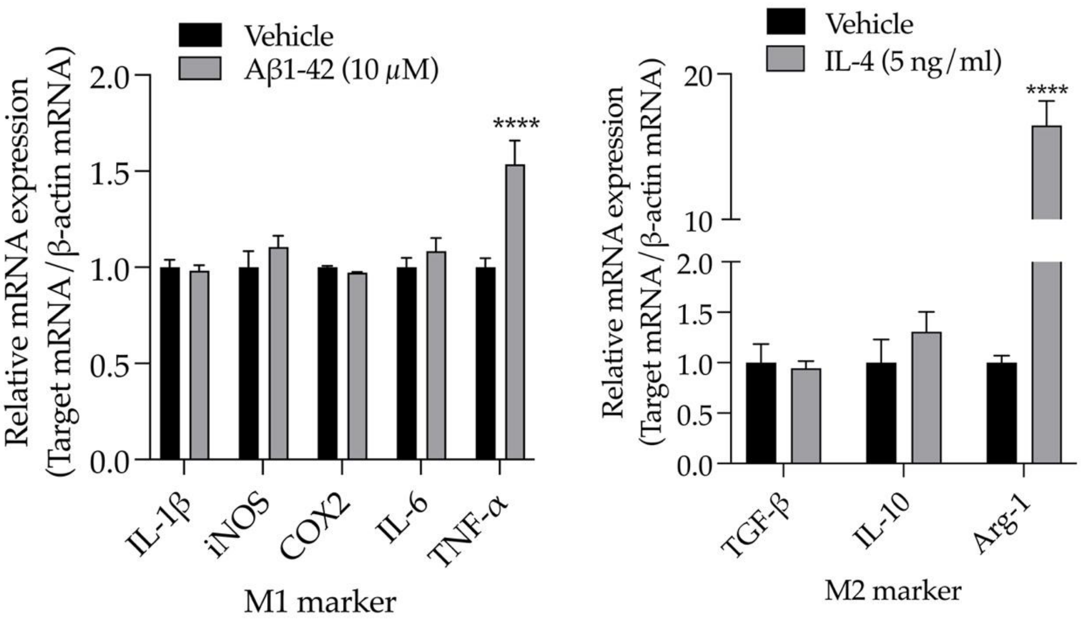

Figure S3. Expression of mRNAs for M1 and M2 markers after stimulation with Aβ-1-42 and IL-4 for 4h. ****$p<0.0001$ compared with vehicle control.
